# A Mobile Analytical Chemistry Workstation with a C4D Sensor for Rapid Detection of Organophosphates Under Field Conditions

**DOI:** 10.3390/s25113517

**Published:** 2025-06-03

**Authors:** Yineng Wang, Xi Cao, Walter Messina, Anna Maria Hogan, Justina Ugwah, Eric Moore

**Affiliations:** 1 Life Science Interface Group, Micro & Nano Systems Centre, Tyndall National Institute, T12 R5CP Cork, Ireland; yineng.wang@tyndall.ie (Y.W.); xi.cao@umail.ucc.ie (X.C.); walter.messina@tyndall.ie (W.M.); annamaria.hogan@ucc.ie (A.M.H.); justina.ugwah@tyndall.ie (J.U.); 2 School of Chemistry, College of Science, Engineering & Food Science, University College Cork, T12 YN60 Cork, Ireland; 3 G18 Rapid Prototyping Lab, Environmental Research Institute, T23 XE10 Cork, Ireland

**Keywords:** mobile analytical workstation, capillary electrophoresis (CE), capacitively coupled contactless conductivity detection (C4D), organophosphate detection, PDMS microfluidic chips, on-site analysis, environmental monitoring

## Abstract

**Highlights:**

- The mobile workstation integrates capillary electrophoresis with C4D on disposable PDMS chips, enabling rapid, sensitive organophosphate detection in the field.
- Optimised injection techniques, voltage settings, and a custom adaptor ensure reproducible separations and robust performance under challenging field conditions.
- The portable system reduces analytical turnaround times relative to laboratory methods, enabling rapid decisions in forensic and environmental scenarios.
- Its cost-effective, user-friendly design supports widespread on-site deployment, boosting emergency response and public safety.

**Abstract:**

Timely detection of organophosphates in outdoor environments remains a critical challenge for forensic and environmental monitoring. Traditional methods often require transporting samples to centralised laboratories, delaying essential response actions. In this study, we present a novel mobile analytical chemistry workstation that integrates capillary electrophoresis (CE) with capacitively coupled contactless conductivity detection (C4D) on low-cost polydimethylsiloxane (PDMS) microfluidic chips, enabling rapid and accurate on-site analysis of organophosphates. The system features a streamlined workflow that includes in-field sample collection, microfluidic analysis, and the wireless transmission of data to a central command centre for immediate decision-making. The detection system demonstrates a linear range of 2.5 mM to 20 mM for dimethyl methylphosphonate (DMMP), with an estimated limit of detection (LOD) of 2.5 mM. We evaluate the feasibility of combining CE and C4D under field conditions, highlighting both the strengths and limitations of this integrated platform.

## 1. Introduction

**Figure 1:**
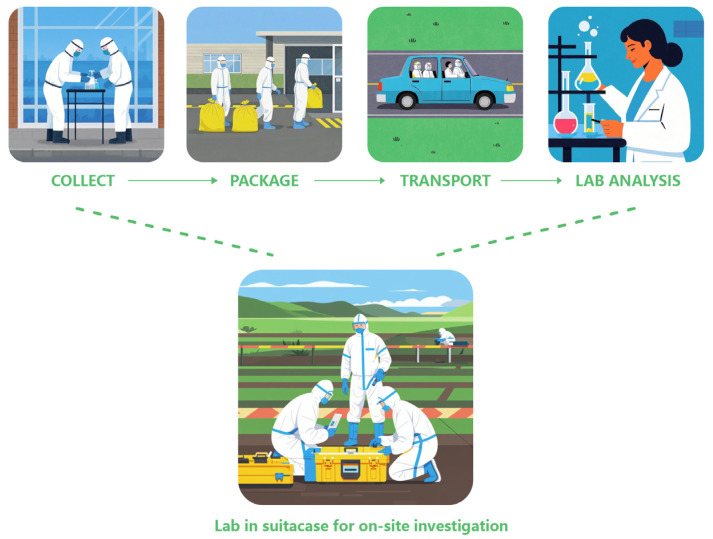
Conceptual illustration of the “lab-in-a-suitcase” workflow.

In on-site investigations, agricultural safety, and environmental monitoring, investigators often require a preliminary identification of a sample’s chemical composition to decide if they require further laboratory analysis or can be discarded. Current on-site chemical speciation instruments, including ion mobility spectrometers, Fourier transform infrared spectrometers, photoionisation detectors, and flame photometric detectors often lack adequate mobility or resolving power, resulting in high false alarm rates or overly selective detection limited to specific compound families [1]. To overcome these limitations, it is crucial to develop complementary on-site chemical detection technologies. These technologies should enable preliminary testing at crime scenes, offering attributes such as low cost and ease of use, especially for operators without specialised chemistry training. Figure 1 presents the conventional analysis workflow, which consists of

- On-site sampling;
- Sample packaging and labelling;
- Transportation;
- Laboratory analysis;

This traditional approach frequently causes delays. The development of an on-site chemical analysis workstation offers a promising solution, enabling real-time analysis of relevant chemicals to enhance management oversight. Furthermore, analysed data could be instantly shared via a cloud server, ensuring accessibility for all relevant units. Administrative teams could leverage this rapid on-site analysis to mitigate hazards from excessive pesticide use.

Organophosphates (OPs) are highly toxic compounds widely used as pesticides and chemical warfare agents. Their acute toxicity, potential for misuse, and environmental persistence present significant risks to public health, food safety, and national security. In laboratory settings, OPs are analysed using various chromatographic techniques, with gas chromatography (GC) and liquid chromatography (LC) being the most prevalent. GC-based analyses typically involve separation using GC coupled with mass spectrometry (GC-MS) [2,3,4] or a flame photometric detector (GC-FPD) [5,6,7]. For LC, high-performance liquid chromatography (HPLC) is commonly paired with UV/Vis detection [8] or mass spectrometry (LC-MS) [9,10].

Capillary electrophoresis (CE) presents a compelling alternative for OP analysis due to its rapid method development, low reagent consumption, short analysis times, operational simplicity, and high separation efficiency [11,12,13,14]. Compared to GC and LC, CE provides distinct advantages, including the elimination of carrier gases and high temperatures required in GC and the use of environmentally friendly aqueous buffers instead of organic solvents used in LC. Additionally, CE utilises electroosmotic flow (EOF) rather than pressure-driven mechanisms, simplifying system design and enhancing suitability for portable, on-site analysis. Numerical studies have previously explored the fluidic behaviour in microchannels, including EOF-driven sample transport and dispersion effects under various conditions [15], but practical implementation under field conditions remains limited.

Miniaturised CE (microchip CE) has been integrated with various optical detection techniques, such as UV/Vis [16], laser-induced fluorescence (LIF) [17], and chemiluminescence (CL) [18]. However, several challenges hinder the widespread adoption of these methods in miniaturised systems. These include the need for high-precision, costly instrumentation that may be too bulky for portable applications, complex sample preparation, and a limited range of detectable analytes.

To address these limitations, we adopted capacitively coupled contactless conductivity detection (C4D) for its seamless integration with CE, cost-effectiveness, simplicity, and portability. C4D is an electrochemical detection technique that uses electrodes to measure analyte conductivity as they migrate through the CE capillary. Its simplicity, low cost, and minimal power consumption make it ideal for integration into disposable microfluidic devices and on-site deployment [19]. Unlike direct-contact electrochemical methods, C4D’s contactless electrode design prevents solution contamination and simplifies operation and maintenance [20,21].

Capacitively coupled contactless conductivity detection (C4D) applications in electrophoresis emerged in the 1980s [22,23,24] and advanced significantly in the 1990s with developments in capillary zone electrophoresis (CZE) [25]. Chip-based electrokinetic separation methods incorporating C4D have gained traction, especially in bioanalytical applications [26]. Advances in electronic integrated circuits over the past decades have further facilitated miniaturisation and integration of C4D into portable analytical devices [16,20,27]. As a result, C4D has emerged as a cost-effective alternative or complement to optical and mass detection techniques in miniaturised portable analytical platforms.

Although research on portable microchip electrophoresis (MCE)-C4D devices remains limited, notable studies have been reported. Ding et al. investigated a poly(dimethylsiloxane) (PDMS) microchip with C4D for detecting alkyl methylphosphonic acids, achieving analysis times under 2 min and detection limits from 1.3 to 4.5 mg/L [28]. Similarly, Duran et al. reported a portable MCE-C4D device for detecting degradation products of OP nerve agents, with detection limits around 10 µg/mL and a linear response of 10–300 µg/mL [29]. In this study, we present a portable analytical platform specifically designed for rapid, on-site OP detection, supporting timely monitoring and response in field conditions.

Compared to widely used lab-based instruments, which typically require complex mixing or pumping systems reliant on gas or liquid pressure, CE uniquely utilises high voltage to generate electroosmotic flow (EOF) as the mobile phase [30,31]. This feature significantly reduces required components, thereby facilitating system miniaturisation and enhancing portability for practical field deployment.

## 2. Materials and Methods

### 2.1. Chemicals

**Figure 2:**
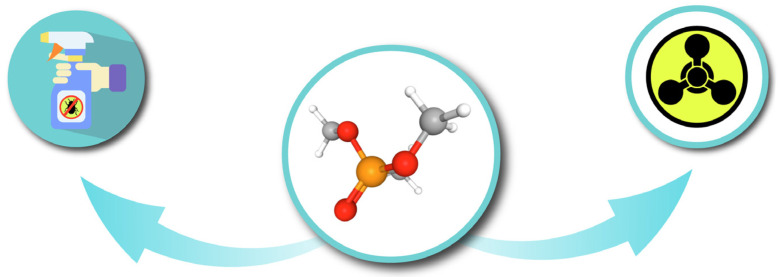
DMMP can be a precursor of pesticides and chemical weapons such as Sarin.

Dimethyl methylphosphonate (DMMP), whose molecular structure is illustrated in Figure 2, is an organophosphorus compound with the chemical formula CH_3_PO(OCH_3_)_2_. Classified as a Chemical Weapons Convention (CWC) Schedule 2 chemical, DMMP is utilised in the production of pesticides and may also serve as a precursor in the synthesis of chemical weapons such as Sarin [32]. In this study, we employed DMMP as the primary analyte due to its low toxicity and its molecular resemblance to pesticides that may be present on crops or in soil.

DMMP is an uncharged (neutral) organophosphorus compound. However, in an electrophoretic system it will be carried through the capillary by the bulk electroosmotic flow (EOF) of the buffer [33]. Because DMMP is a neutral, moderately hydrophobic molecule, introducing a hydrophobic “stationary phase” or pseudostationary phase can enable its separation via hydrophobic interactions. In capillary electrochromatography (CEC) and micellar electrokinetic chromatography (MEKC), for example, neutral analytes are resolved by differential partitioning into hydrophobic phases (such as octadecylsilane coatings or surfactant micelles) while being transported by EOF [34]. Following this principle, using a hydrophobic medium greatly enhances the separation of DMMP. Polydimethylsiloxane (PDMS), the material commonly used for microchip channels, has a hydrophobic surface and can itself act as a sort of stationary phase through hydrophobic absorption of analytes [35]. In fact, native PDMS microchannels are known to strongly absorb hydrophobic molecules, which can retard their movement relative to the bulk flow [35]. Here, we exploit PDMS as a hydrophobic stationary phase to achieve the separation of the neutral, nonpolar DMMP via hydrophobic interactions in a microchip CE system.

### 2.2. Lab-on-Chip Device Fabrication

#### 2.2.1. Pick-Up Electrode Cell Fabrication

**Figure 3:**
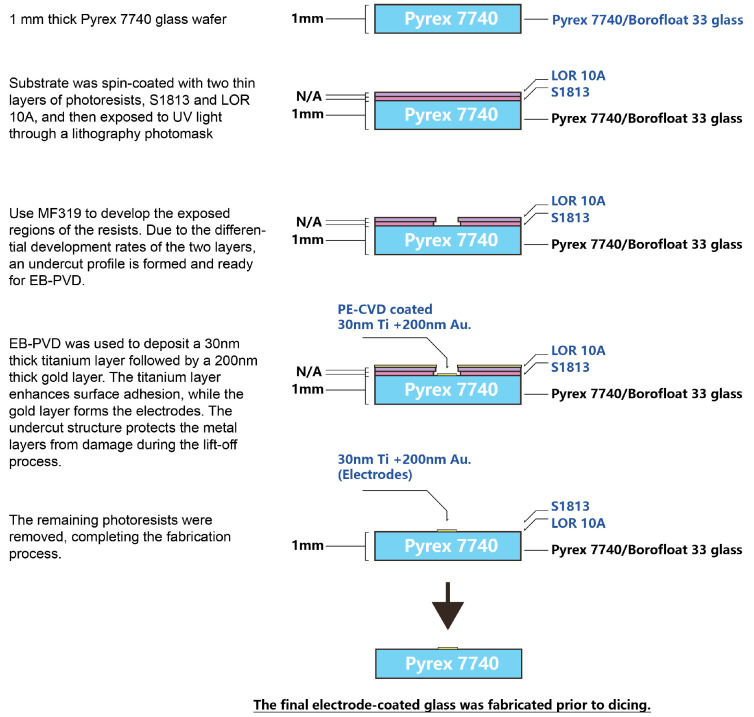
Fabrication process for the pick-up electrode cell on glass.

**Figure 4:**
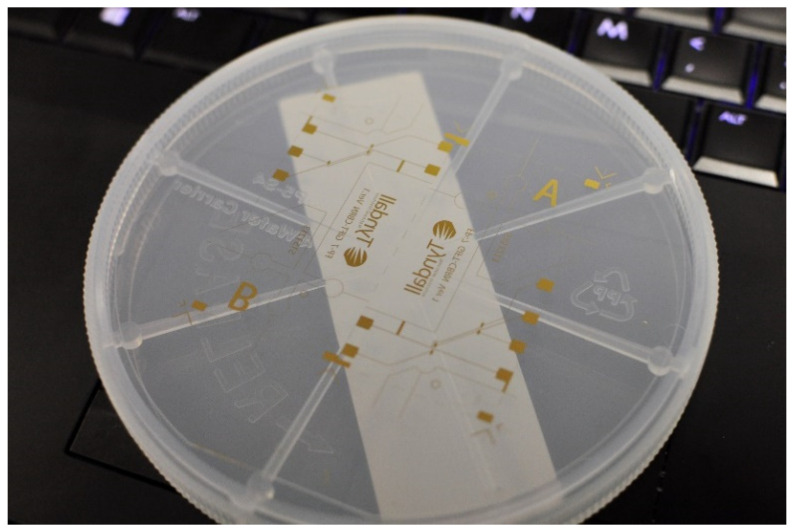
The fabricated pick-up electrode cell on glass (top side, showing metal layer).

The gold pick-up electrode cell, fabricated from a 1 mm thick Pyrex 7740 glass wafer substrate, is commercially available from University Wafers (Boston, MA, USA). Figure 3 illustrates the detailed fabrication process for the gold pick-up electrode cell substrate. A classic “lift-off” lithography technique was employed to create an undercut profile. First, a layer of S1813 photoresist was spin-coated and coated onto the substrate, followed by a second layer of LOR10A photoresist. The UV exposure time was one second. Due to the different development rates of these two photoresists, the developer MF319 removed more material from the S1813 layer than from the LOR10A layer, resulting in an undercut that facilitated the lift-off of the subsequent metal deposition layer. Electron Beam Physical Vapour Deposition (EB-PVD) was then used to deposit a 30 nm titanium layer to enhance surface adhesion, followed by a 200 nm gold layer on top of the titanium. Figure 4 presents the pick-up electrode cell device. Thus, the electrode cell body was successfully fabricated on the glass wafer substrate.

#### 2.2.2. Polydimethylsiloxane (PDMS) Device Fabrication Processes

**Figure 5:**
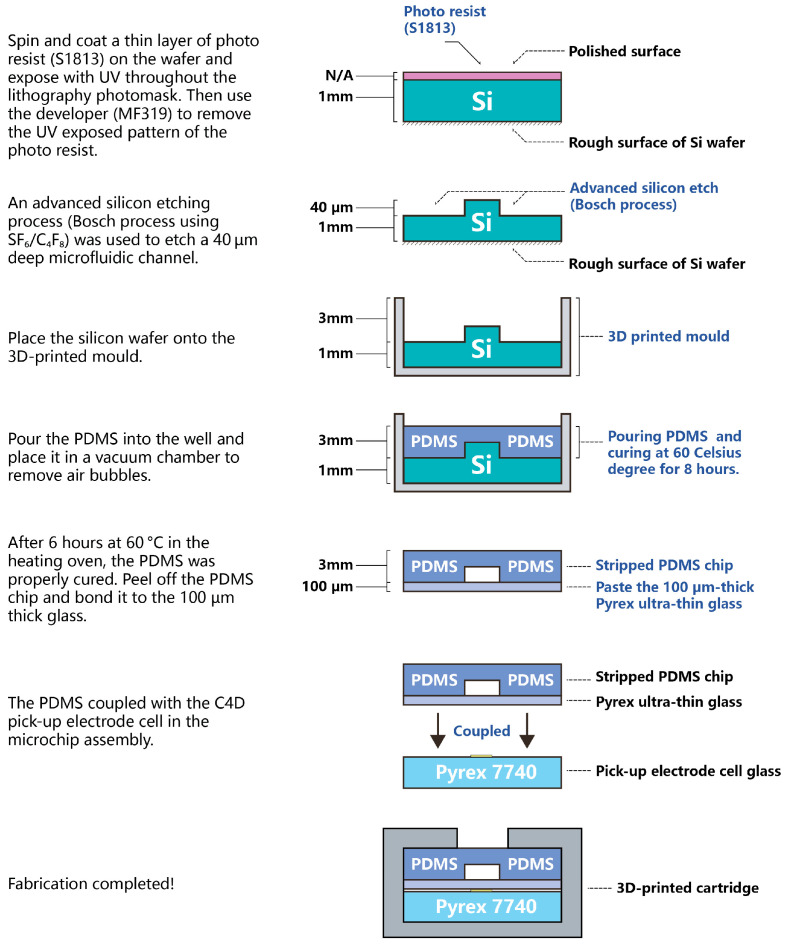
The PDMS microchip fabrication process.

The PDMS microchip device represents an iteration of our previous research [31,36]. Figure 5 illustrates the PDMS microchip fabrication process. The mould for the microchip was produced using a 1 mm thick, a 100 mm diameter P-type silicon wafer (1-0-0), which was polished on one face and left rough on the other. A conventional lithographic process was employed on the silicon wafer, using a spin-coating technique to define the microfluidic channel locations. A thin layer of S1813 photoresist was applied to the substrate and then exposed to UV light through a lithography photomask. Subsequently, the MF319 developer was used to remove the UV-exposed regions of the photoresist. An Advanced Silicon Etching (ASE) technique with (SF6/C4F8 with 5:1 ratio) was then employed to etch microfluidic features to a depth and width of 40 µm ridge into the polished surface of the silicon wafer. After etching, the remaining photoresist was removed with an organic solvent.

**Figure 6:**
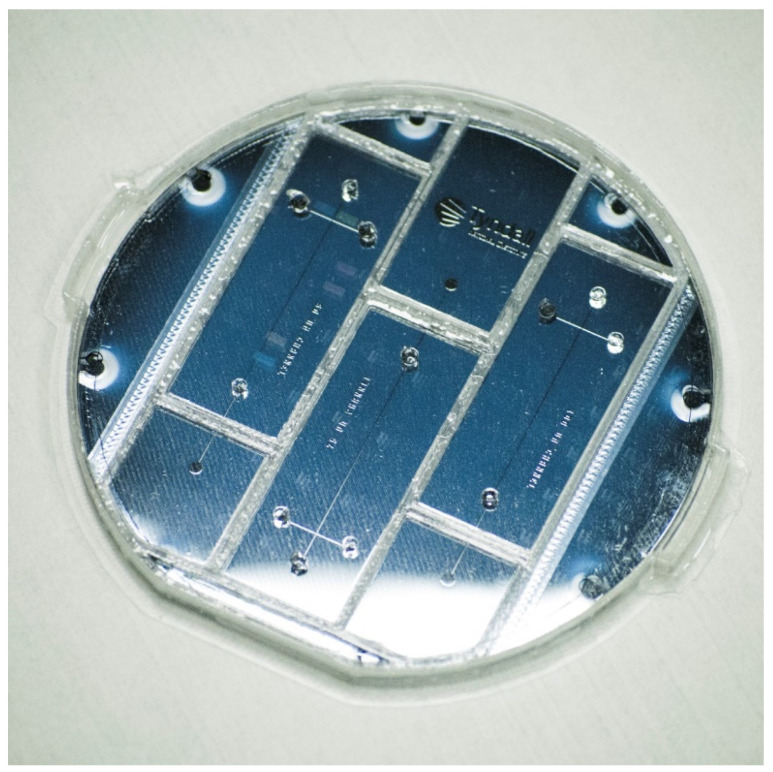
The PDMS microchip in the 3D-printed mould.

The etched silicon wafer was then placed in a custom-designed, 3D-printed mould (shown in Figure 6) that was integrated into the cartridge to align each chip with its respective reservoirs. Liquid PDMS was poured over the mould and cured in a thermal oven at 60 °C for 6 h. Once cured, the PDMS microchip was carefully peeled off and bonded to a thin glass substrate (Model 0, 50 mm × 22 mm, 0.08–0.10 mm thick; Bio-cover-glass, Agar Scientific Ltd., Rotherham, UK). Finally, the PDMS microchip was aligned and coupled with the pick-up electrode cell. The effective separation channel length was set to 28 mm (from the “Double-L” intersection to the pick-up electrode cell), based on our experience.

#### 2.2.3. PDMS Microchip Packaging (Assembling)

**Figure 7:**
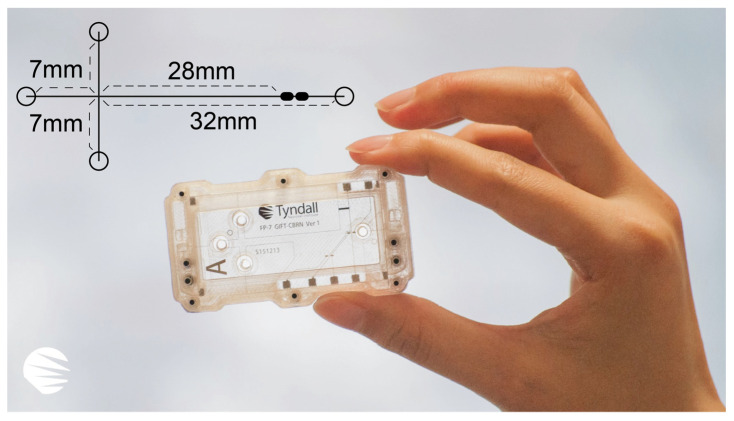
The PDMS microchip assembly with microchannel lengths indicated. Branch channels are 7 mm, the main separation channel is 32 mm long, and the effective separation length is 28 mm.

Figure 7 illustrates the PDMS microchip, housed in a fully 3D-printed polymer cartridge that encloses all essential components, including the pick-up electrode cell glass and the microchip itself. The upper section of the cartridge also provides additional reservoir volume. PDMS is one of the most widely used polymers in microdevice fabrication, thanks to its biocompatibility and ease of manufacture [35]. Moreover, the PDMS microchip assembly is considerably more cost-effective and is designed to be disposable.

### 2.3. Microchip Adaptor Platform Design

As the gold-plated copper electrode was unsuitable for high-voltage (HV) applications, we developed a Pt wire electrode integrated with a custom-designed microchip adaptor platform. This platform is designed to house the microchip assembly and facilitate analysis, featuring an interlock switch that ensures high voltage can only be activated when the hatch is securely closed. This safety mechanism safeguards the user.

**Figure 8:**
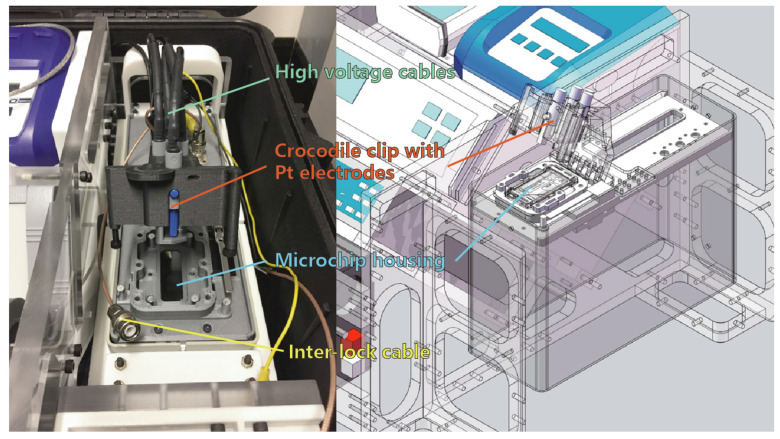
Prototype of the microchip adaptor platform and its CAD model.

Figure 8 shows the prototype of the microchip adaptor platform. Four short platinum wires are secured by cylindrical crocodile clips mounted on the upper hatch of the platform. Each clip is equipped with a standard 4 mm “bullet head” connector, which attaches the HV coaxial cables to the HVS 448 sequencer instrument (LabSmith HVS448-6000D-LC High Voltage Sequencer, LabSmith, Inc., Livermore, CA, USA). When the hatch assembly is closed, the four platinum wires simultaneously insert into the microchip reservoirs, delivering high voltage to drive the electroosmotic flow (EOF) within the fluidic channel.

Additionally, the microchip platform incorporates four magnets to secure the microchip in place. The complete microchip adaptor module slides into the right side of the suitcase system and can be quickly replaced to accommodate future upgrades, such as the integration of an optical sensing system.

### 2.4. Suitcase System Integration

**Figure 9:**
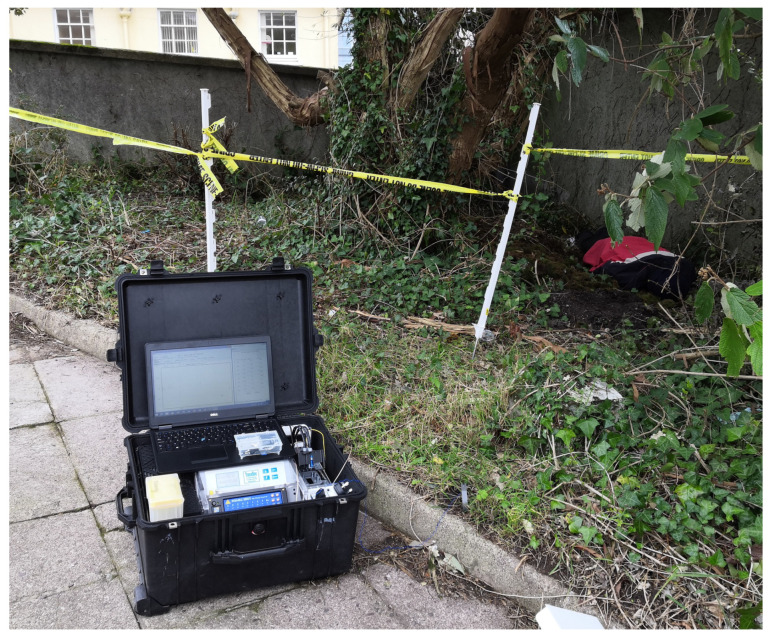
The suitcase system prototype during a field test.

The suitcase system (shown in Figure 9) comprises the following sub-module instruments: the TraceDec C4D detector (sourced: TraceDec^®^—Contactless Conductivity Detector, Innovative Sensor Technologies GmbH, Strasshof an der Nordbahn, Austria), the LabSmith HVS 448 high-voltage sequencer, three Sunon DP200 cooling fans (sourced: Radionics Ltd., Dublin, Ireland), three LRP 7.4 V 2-cell lithium polymer batteries (connected in series) & the LRP pulsar balanced battery charger (sourced: LRP GmbH, Schorndorf, Germany), the Mascot 300 W DC-AC car power inverter (sourced: Radionics Ltd., Dublin, Ireland). All these modules are neatly and securely mounted on a PMMA-machined frame. These components have been arranged within a custom-made frame to create a robust, professional instrument suitcase. Hence the name “suitcase system”. The system’s interface is a Microsoft Surface Pro tablet (provided by UCC department of IT Service) running the InfoHub software v0.1 developed by our project partner, AnalyzIQ Ltd. (Cahercrin, Athenry, Galway, Ireland.). This setup facilitates real-time detection and data sharing: Once the suitcase system detects a hazardous substance, the results can be immediately transmitted to other units via the InfoHub software. Equipped with all essential instruments for standalone on-site analysis and supported by onboard batteries, the system can function as an independent analysis unit deployed by the operational team.

To ensure adequate power for the sub-modules and maintain optimal functionality, a custom power management system was developed. A DC-to-AC converter was designed to provide a standard 230 V AC output to the C4D and HV sequencer instruments. Since most airlines restrict battery capacity to below 160 Wh, a six-cell Li-Po (lithium polymer) battery pack with a total capacity of 155.4 Wh was used, operating within a voltage range of 21 V (discharged limit) to 25.2 V (fully charged limit). The battery pack comprises three two-cell batteries (LRP GmbH, Germany; 7000 mAh, 7.4 V Li-Po) connected in series. A balanced charger (LRP GmbH, Germany, Polsar Li-Po battery charger) has been integrated into the system, ensuring that each battery cell is charged and monitored individually. This balanced charging configuration maximises battery life and capacity.

#### 2.4.1. Instruments

The TraceDec^®^ C4D detector, when combined with capillary electrophoresis or chromatography, provides a highly sensitive detection method for various analytical applications. It offers amplitude settings of 0 dB, −6 dB, and −12 dB, corresponding to output voltages of 36 V, 18.61 V, and 9.31 V, respectively. Activation frequency options include medium, high, and 2× high, which correspond to 77 kHz, 153.5 kHz, and 307 kHz. Control and data acquisition are managed through software on a Surface Pro tablet, with an optional feature for data sharing via the InfoHub software (customised and developed by our team). The home-built power management system supplies 230 V AC directly to this detector as a sub-module of the suitcase system, and its output and input are directly connected to the microchip adaptor platform.

The LabSmith HVS448-6000LC (low-current version) high-voltage sequencer provides eight high-voltage channels with programmable sequencing output. Each channel is capable of supplying and sensing up to ±3.0 kV and ±6 mA, with a 16-bit current sensing resolution. Control is managed through software on the Surface Pro tablet. The home-built power management system provides 230 V AC directly to this sequencer as a sub-module of the suitcase system, and its high-voltage output is directly connected to the microchip adaptor platform.

#### 2.4.2. Chemical Consumables

- DMMP: Dimethyl methylphosphonate, purum ≥ 97.0%, Sigma-Aldrich Ireland (Prod. No. 64258-250ML).
- MES: 2-(N-morpholino)ethanesulfonic acid (MES), ≥99% (titration) (Sigma-Aldrich/Merck, SKU: 475893-500GM), purchased from Sigma-Aldrich/Merck Ireland.
- Histidine: L-Histidine, (S)-2-Amino-3-(4-imidazolyl)propionic acid, ≥99.5% (NT) (Sigma-Aldrich/Merck, SKU: 53319-25G), purchased from Sigma-Aldrich/Merck Ireland.
- Buffer preparation: MES/Histidine buffer was prepared at concentrations of 12.5 mM and 25 mM. The pH was adjusted to 5.73 using a Metrohm 654 pH meter equipped with a microelectrode (Metrohm 6.0232.100).
- Sample preparation: Various concentrations of the analyte DMMP (2.5 mM, 5 mM, 10 mM, and 20 mM) were prepared in the MES/Histidine buffer.

### 2.5. Method Development

#### 2.5.1. The Double-L Injection

**Figure 10:**
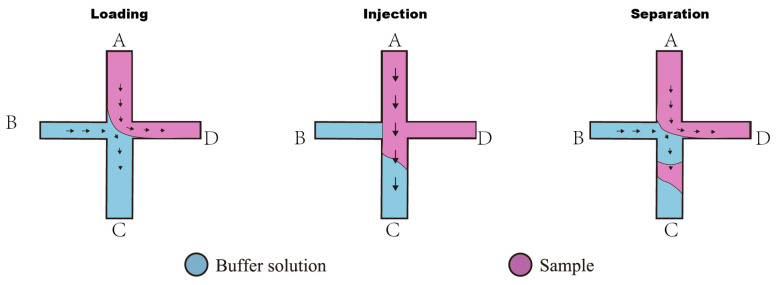
A “Double-L Injection” technique on the microchip electrophoresis application (Uppercase letters indicate each corresponding reservoir, and the black small arrows indicate the direction of EOF).

Figure 10 shows the injection technique divided into three phases: loading, injection, and separation. Table 1 and Table 2 show the voltage setups during the test.

- Loading Phase: The sample is added to reservoir A and driven toward the junction (reservoir D) by EOF, positioning a sample plug at the injection intersection. Meanwhile, a voltage applied between reservoirs B and C maintains a fresh buffer flow.
- Injection Phase: A brief (approximately 1 s) voltage pulse is applied to reservoir A only, injecting the sample plug into the separation channel.
- Separation Phase: The voltages are returned to the loading configuration (voltage reapplied at B), and EOF carries the sample plug through the channel. Within minutes, distinct peaks appear, indicating a successful separation.

#### 2.5.2. System Peak Optimisation

**Figure 11:**
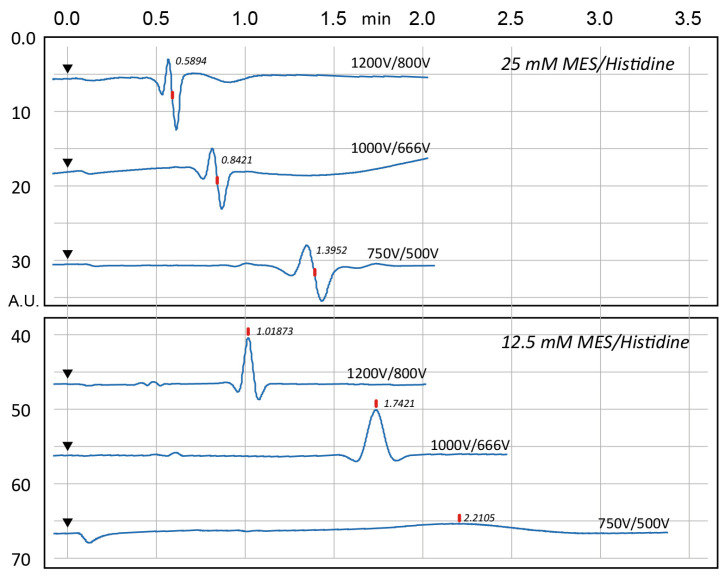
The result shows the system peak when running the MES/Histidine buffer, comparing 12.5 mM and 25 mM MES/Histidine buffer backgrounds. Red dots indicate the system peak migration time, and black triangles mark the sample injection.

Figure 11 shows that the characteristic “system peak” arises from the sudden application of high voltage—the instrument output jumps from 0 to hundreds of volts to initiate electroosmotic flow (EOF). This is a common phenomenon observed when CE and C4D detection are combined. Two buffer concentrations (25 mM and 12.5 mM) and three voltage configurations were tested. At 25 mM buffer concentration, the system peak exhibited a distorted (“twisted”) shape; when the buffer was diluted to 12.5 mM, this peak distortion was greatly diminished. The system peak was also observed to migrate more slowly with the lower (12.5 mM) buffer, as expected. Additionally, reducing the applied voltages led to a smaller system peak but at the cost of longer separation times. Based on these findings, a voltage configuration of 750 V (reservoir B) and 500 V (reservoir A) with a 12.5 mM MES/Histidine buffer was selected as the optimal condition for subsequent tests.

## 3. Results

A test was conducted using the suitcase system to determine the limit of detection (LOD) for DMMP under the optimised conditions. A fresh MES/Histidine buffer was prepared at a concentration of 12.5 mM. DMMP solutions were then prepared at various concentrations (2.5 mM, 5 mM, 10 mM, and 20 mM). The high-voltage sequence was set as follows: reservoir A = 500 V; reservoir B = 750 V; reservoirs C and D = GND. The activation frequency was set to 153.5 kHz, with an amplitude of 18.61 V.

**Figure 12:**
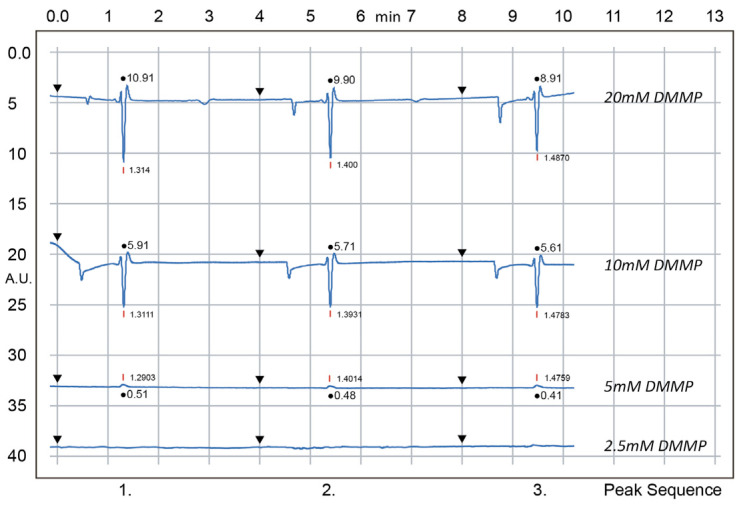
Electropherograms for DMMP at various concentrations (2.5 mM, 5 mM, 10 mM, and 20 mM) in 12.5 mM MES/Histidine buffer were obtained with the suitcase system. Red bars (with numeric labels) indicate the DMMP peak migration times; black circles show peak areas (with values); and black triangles mark the sample injection.

**Figure 13:**
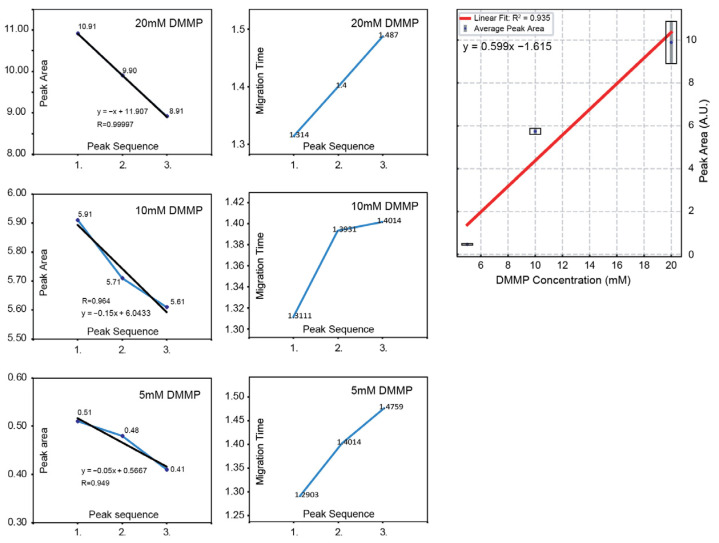
Plots of DMMP peak area (**left**) and migration time (**right**) under varying conditions/injections.

The data (Figure 12 and Figure 13) show that three replicate injections were performed for validation, with a total cycle time of 4 min per run. Overall, the baselines remained flat across all concentration levels, while both the peak area and peak height increased in proportion to the analyte concentration. However, within each concentration run, a slight decrease in peak area was observed over three successive injections, accompanied by a gradual increase in migration time. This effect is likely due to the slight depletion of buffer in the reservoir over multiple injections (because EOF-driven flow can lower the buffer level, affecting EOF rate).

## 4. Discussion

The specificity of the separation method in the presence of structurally similar hydrophobic compounds is an important consideration. As this study presents a proof-of-concept prototype tested under realistic field conditions, we acknowledge that the current system does not yet incorporate highly selective buffer chemistries such as surfactant-modified phases, derivatisation strategies, or micellar systems commonly used for advanced chemical discrimination. Nonetheless, our experimental results demonstrated consistent retention of DMMP, which we attribute to hydrophobic interactions with the PDMS microchannel surface. This behaviour suggests that the PDMS channel may act as a pseudo-stationary phase, retarding certain analytes through surface adsorption. While this mechanism may not provide high specificity when multiple structurally similar hydrophobic compounds are present, it supports the feasibility of neutral molecule separation via PDMS–analyte interaction under EOF-driven conditions. Future development will focus on improving selectivity—potentially through micellar electrokinetic chromatography (MEKC), alternative coatings, or buffer additives.

Compared to previously reported portable MCE–C4D systems, our detection limit of 2.5 mM for DMMP (equivalent to approximately 310 mg/L) is higher than the sub-millimolar levels achieved by prior studies for ionic organophosphates—for instance, Ding et al. [28] reported LODs of 1.3–4.5 mg/L for alkyl methyl-phosphonic acids, while Duran et al. [29] achieved LODs around 10 µg/mL for degradation products of nerve agents. These lower values were attainable due to the direct detection of charged species. In contrast, DMMP is a neutral molecule detected indirectly via shifts in conductivity, resulting in inherently lower sensitivity.

In this study, we present a mobile analytical chemistry workstation that integrates capillary electrophoresis (CE) with capacitively coupled contactless conductivity detection (C4D) on low-cost polydimethylsiloxane (PDMS) microfluidic chips. The system demonstrated its capability for rapid on-site analysis of organophosphates (OPs), offering a streamlined workflow that includes in-field sample collection, microfluidic analysis, and wireless data transmission for immediate decision-making.

The key findings indicate that the system has a linear detection range of 2.5 mM to 20 mM for DMMP and an estimated limit of detection (LOD) for DMMP of 2.5 mM. These results support the potential of this mobile workstation for emergency response, environmental monitoring, and public health protection, where traditional “on-site sampling to lab analysis” methods are often too slow and resource-intensive.

The implications of this work suggest that integrating CE and C4D on a compact platform can make organophosphate detection more accessible, plug and play, cost-effective, and portable. This system could be applied in a range of scenarios, including emergency response, environmental monitoring, and public health safety. However, there are limitations to this approach. The simplicity of the microchip design, while contributing to low cost and ease of use, also presents certain stability challenges (e.g., slight shifts in peak area and migration time over successive runs), as indicated by the gradual increase in migration time and slight variability (decrease) in peak area observed during testing. Further work will be required to optimise the system’s stability and enhance its performance in various environmental conditions.

Although the current detection limit (2.5 mM) is higher than that of laboratory-grade systems, this platform demonstrates the capability for real-time, field-deployable screening. Future developments will focus on enhancing sensitivity by integrating UV detection within the chip adaptor platform for “C4D and UV” dual-mode analysis and peak confirmation.

## Figures and Tables

**Table 1:** Three candidate reservoir voltage setups are in the loading and separation stages.

Reservoir	A	B	C	D
Setup 1	0.8 kV	1.2 kV	GND	GND
Setup 2	0.67 kV	1 kV	GND	GND
Setup 3	0.5 kV	0.75 kV	GND	GND

**Table 2:** Three candidate reservoir voltage setups are in the injection stages.

Reservoir	A	B	C	D
Setup 1	0.8 kV	GND	GND	GND
Setup 2	0.67 kV	GND	GND	GND
Setup 3	0.5 kV	GND	GND	GND

## Data Availability

The data presented in this study are available on request from the corresponding author.

## Abbreviations

The following abbreviations are used in this manuscript:

AC	Alternating Current
ASE	Advanced Silicon Etching
CAD	Computer-Aided Design
CE	Capillary Electrophoresis
C4D	Capacitively Coupled Contactless Conductivity Detection
CWC	Chemical Weapons Convention
DC	Direct Current
DMMP	Dimethyl Methylphosphonate
EB-PVD	Electron Beam Physical Vapor Deposition
EOF	Electro-Osmotic Flow
GND	Ground
HVS	High-Voltage Sequencer
Li-Po	Lithium Polymer
MES	2-(N-Morpholino) Ethane Sulfonic Acid
PDMS	Poly-Di-Methyl-Siloxane
PMMA	Polymethyl Methacrylate
UV	Ultra-Violet
